# Comparison of Antigen Conjugation to a Peptidic Carrier or to Bovine Serum Albumin in the Serodiagnosis of Canine Visceral Leishmaniasis via Suspension Array Technology

**DOI:** 10.3390/antib14040103

**Published:** 2025-12-04

**Authors:** Thais Stelzer Toledo, Pauline Martins Cunha, Josué da Costa Lima-Junior, Monique Paiva De Campos, Alinne R. S. Renzetti, Fabiano Borges Figueiredo, Fernanda Nazaré Morgado, Renato Porrozzi, Fatima da Conceição-Silva, Marta de Almeida Santiago, Paula Mello De Luca

**Affiliations:** 1 Laboratory of Immunoparasitology, Oswaldo Cruz Institute (IOC), Fundação Oswaldo Cruz (Fiocruz), Rio de Janeiro 21040-900, Brazil; 2 Postgraduate Program in Parasitic Biology, Oswaldo Cruz Institute (IOC), Fundação Oswaldo Cruz (Fiocruz), Rio de Janeiro 21040-900, Brazil; 3 Professional Master’s in Immunobiological Technology, Immunobiological Technology Institute (Bio-Manguinhos), Fundação Oswaldo Cruz (Fiocruz), Rio de Janeiro 21040-900, Brazil; 4 Leishmaniasis Reference Laboratory, Carlos Chagas Institute (ICC), Fundação Oswaldo Cruz (Fiocruz), Curitiba 81350-010, Brazil; 5 Postgraduate Program in Clinical Research in Infectious Diseases, Instituto Nacional de Infectologia Evandro Chagas (INI), Fundação Oswaldo Cruz (Fiocruz), Rio de Janeiro 21040-900, Brazil; 6 Cellular Biology Laboratory, Carlos Chagas Institute (ICC), Fundação Oswaldo Cruz (Fiocruz), Curitiba 81350-010, Brazil; 7 Protozoology Laboratory, Oswaldo Cruz Institute (IOC), Fundação Oswaldo Cruz (Fiocruz), Rio de Janeiro 21040-900, Brazil

**Keywords:** canine visceral leishmaniasis, immunodiagnostic, non-magnetic beads, suspension array technology, cytometric bead array, bovine serum albumin

## Abstract

**Backgroud/Objectives**: Canine Visceral Leishmaniasis (CVL), caused by *Leishmania infantum*, is a significant public health concern due to dogs serving as reservoirs for human infection. An accurate and rapid diagnostic method to distinguish symptomatic and asymptomatic CVL from healthy and vaccinated animals is essential for controlling canine and human disease. Developing innovative antibody detection techniques and exploring new antigens are essential for enhancing CVL testing efficiency. Our study focuses on a multiplex flow cytometry technique to detect *Leishmania*-specific antibodies in canine serum. This involved conjugating small peptides with carrier proteins or peptide tags, sequences designed to facilitate bead coupling. **Methods**: A peptide from the *L. infantum* A2 protein was coupled to beads in three forms: unconjugated, conjugated with BSA, and conjugated with a C-terminal β-alanine–lysine (x4)–cysteine TAG. This TAG was previously designed to enhance peptide solubility, improve binding efficiency, and provide functional groups for covalent attachment to the beads, ensuring stable immobilization in the multiplex assay. **Results**: Our results suggest that the multiplex approach shows promise as a rapid serological test for CVL, particularly with TAG-conjugated peptides, which optimize bead coupling. However, peptide/BSA conjugation revealed anti-BSA antibodies in samples from healthy and CVL dogs. **Conclusions**: In conclusion, our findings highlight the potential of multiplex methodologies to enhance CVL diagnostics and caution against using BSA as a bead coupling agent in serological tests for canine samples due to its impact on test specificity and sensitivity.

## 1. Introduction

Leishmaniasis are a group of anthropozoonotic diseases caused by protozoan parasites belonging to the genus *Leishmania*, primarily affecting populations in the intertropical regions of the planet [1]. These parasites are transmitted to vertebrate hosts through the bite of female sandflies, mainly of the genera *Phlebotomus* and *Lutzomyia* [2]. In humans, leishmaniasis manifests itself in three main forms: cutaneous leishmaniasis (most common), mucocutaneous leishmaniasis (with chronic progression and lower incidence) and visceral leishmaniasis (VL), also known as kala-azar. VL is a systemic and potentially fatal infection, considered by the World Health Organization (WHO) as one of the most significant parasitic diseases worldwide [3,4].

In VL, visceral organs are parasitized, causing systemic symptoms like prolonged fever, spleen and liver enlargement, weight loss, anemia, pancytopenia, and high antibody titers. Lymphadenopathy and skin darkening may appear in advanced cases. Comorbidities, including bacterial and HIV infections, can alter symptoms and hinder early diagnosis [5].

In the *Leishmania* life cycle, canids play a central role as reservoirs of the parasites. VL exhibits two significant epidemiological-biological cycles: an anthroponotic cycle and a zoonotic cycle. The anthroponotic cycle is primarily documented in India [6]. The zoonotic cycle is predominant in North Africa, Asia, the Mediterranean, and South America, notably in Brazil. In Central Africa and India, VL is associated with *Leishmania donovani*, while *L. infantum* is responsible for VL in the Americas, Central Asia, China, the Middle East, and the Mediterranean [5,7].

Canine visceral leishmaniasis (CVL), caused by *L. infantum*, is a multisystemic disease that progresses slowly and chronically, displaying a wide spectrum of clinical signs, or sometimes remaining asymptomatic [8,9]. Considering the diversity of potential signs and symptoms, which are also common to other diseases, and the non-specific changes observed in blood counts and renal or hepatic function tests, clinical diagnosis of CVL is challenging. Additionally, animals may remain asymptomatic for extended periods or develop symptoms months or years after infection, thereby increasing their role as reservoirs and sources of human infection [4,8]. Taking these complexities into account, laboratory diagnosis is essential for CVL control. It enables early decision-making regarding pharmacological intervention (treatment) and the implementation of transmission-blocking measures, such as relay collars, screens in homes and kennels, or even euthanasia of infected animals in certain control protocols. In Brazil, serological tests are the preferred methods for diagnosing CVL, in accordance with the protocol outlined by the Brazilian Ministry of Health. The initial screening test is the TR-DPP (Dual Path Platform), followed by the ELISA, which serves as the confirmatory test [10]. Although this protocol offers practicality and cost-effectiveness, several studies have highlighted performance issues, including cross-reactions with common infectious agents in dogs (such as *Trypanosoma cruzi*, *L. braziliensis*, *Sporothrix* sp., *Ehrlichia canis* and *Babesia canis*). Additionally, there is reduced diagnostic accuracy in asymptomatic or oligosymptomatic animals in endemic areas. Furthermore, false-positive diagnoses can occur in vaccinated animals due to interference of the humoral immune response induced by the vaccine with the serological diagnosis of CVL [11].

The key role of serological methods in epidemiological studies and CVL detection highlights the importance of enhancing and developing new diagnostic tests. These innovative tests must provide precise and practical differential diagnosis of CVL within a population, including healthy animals, whether vaccinated or not, as well as oligo- and asymptomatic dogs. Additionally, these tests should be designed to minimize cross-reactivity with other diseases, thereby reducing the likelihood of cross-reactions in the currently employed tests.

Flow cytometry, initially developed for the examination of whole cells, organelles, or nuclei [12], has evolved to encompass the assessment of biological reactions on solid fluorospheres via suspension array technology (SAT) [13,14,15]. SAT employs symmetrically labeled microspheres with dual fluorescent dyes internally. These microspheres can be linked to specific capture reagents, including protein antigens. Distinguishable by their spectral addresses, the microspheres can be combined after coupling to various antigens (Ags), facilitating multiplex assays. This enables swift screening of numerous Ags using minimal plasma volume. Comparative to ELISA, SAT has demonstrated equivalent results in quantifying antibodies (Abs) against tetanus, diphtheria, *Haemophilus influenzae* type B [16], pneumococcal capsular polysaccharides [17,18], and *Neisseria meningitidis* [19]. Moreover, SAT matches or exceeds ELISA’s sensitivity in simultaneously detecting Abs to ten mouse viral and microbial pathogens [20], boasting superior reproducibility and an extended dynamic range [21,22]. Consequently, SAT is emerging as a pivotal technique in immunological investigations.

Numerous serological tests, such as singleplex ELISA and the multiplex Luminex assay, make use of recombinant protein antigens [23,24,25]. Although these tests effectively assess antibody responses, utilizing recombinant protein antigens containing multiple immune epitopes presents limitations compared to employing individual immune epitope peptides within the antigen structure. The incorporation of individual peptide immune epitopes allows for a more precise assessment of individual antibody responses, differentiating, for example, recent and past infection [26] and, in the case of CVL, would make it possible to identify symptomatic and asymptomatic infected dogs and differentiate vaccinated individuals from CVL+ ones. Nonetheless, transitioning from peptides in ELISA to an all-peptide bead-based SAT assay presents challenges, primarily stemming from the difficulties associated with achieving consistent coupling of certain small peptides to the beads.

It has been suggested that peptides with molecular weights below 3000 g/mol tend to exhibit suboptimal binding to microspheres [27,28]. The use of small peptides linked to BSA demonstrated promising results in a multiplex SAT assay developed for the diagnosis of human malaria caused by *Plasmodium falciparum*, reveling no significant disparities among the results generated by the various peptides under examination [27]. On the other hand, in another study involving *P. falciparum* diagnosis, 15 peptides complexed with bovine serum albumin (BSA) were recognized by the sera of tested individuals, but a markedly low responses to one of the peptides was observed, suggesting potential alterations in its antigenicity due to its conjugation with BSA [28]. More recently, a study accomplished peptide-microsphere conjugation by employing a peptide tag (“TAG”) comprising beta-alanine-lysine(x4)-cysteine attached to the C-terminal region of each peptide. This innovative approach yielded highly sensitive results in detecting *P. falciparum* specific IgM and IgG antibodies in the sera of infected individuals [29].

Given the critical need for rapid diagnosis of CVL to effectively manage the disease in endemic regions, the present study aims to evaluate a multiplex assay utilizing carboxylated microspheres (Cytometric Beads Array—CBA) coupled with previously described VQ34 peptide [9] predicted from *L. infantum* A2 protein sequence (GenBank: GQ290460). This approach seeks to enhance the speed and sensitivity of serological CVL diagnosis.

## 2. Materials and Methods

### 2.1. Ethical Statement

The experimental protocols of this study were approved by the Ethical Committee for the Use of Experimental Animals (CEUA) of Instituto Oswaldo Cruz—IOC/FIOCRUZ (healthy and vaccinated animals—Protocol L-45/2015) and of Fundação Oswaldo Cruz—FIOCRUZ (naturally infected animals—Protocol LW-4/17).

### 2.2. Canine Samples

The study utilized serum samples from 6 healthy dogs residing in non-endemic regions for CVL, along with samples from 10 dogs naturally infected with *L. infantum* (5 with symptomatic CVL and 5 asymptomatic). The healthy dogs were all of known history, with defined breeds, dwell in individual pens, and received balanced nutrition. Additionally, they underwent regular deworming and vaccinations against common canine infections. Following clinical examination and negative serological and parasitological tests for *Leishmania*, these dogs were immunized with the Leish-Tec^®^ vaccine (Lot# 034/14—Hertape Calier Saúde Animal^®^, Belo Horizonte, Brazil) and were monitored for up to one year. Blood samples were collected for serum analysis at the initial evaluation (prior to vaccination) and one year after completing the manufacturer-recommended immunization protocol (consisting of three doses with a 21-day interval for dogs aged four months or older). Veterinarians observed the animals for up to 48 h post-immunization to detect any potential vaccine-related adverse effects. Throughout the study period (comprising a one-year follow-up and vaccination), the animals did not receive any other vaccines or medications.

The ten dogs naturally infected with *L. infantum* were from the CVL-endemic region of Barra Mansa (Rio de Janeiro, Brazil). They tested positive in both the DPP^®^ screening test and the EIE-leishmaniasis confirmatory (EIE-CL) ELISA kit (Biomanguinhos/Fiocurz, Rio de Janeiro, Brazil). Among them, five dogs exhibited symptoms of CVL, while the remaining five were asymptomatic. Parasitological tests yielded positive results for all dogs, irrespective of symptomatic status.

The experimental procedures in this study received approval from the Ethical Committee for the Use of Experimental Animals (CEUA) at Fundação Oswaldo Cruz—FIOCRUZ for naturally infected animals (Protocol LW-4/17) and from the Instituto Oswaldo Cruz—IOC/FIOCRUZ for healthy and vaccinated animals (Protocol L-45/2015).

### 2.3. Antigens

A2 protein VQ34 peptide (In silico Prediction and Synthesis)—The in silico prediction of the VQ34 peptide was performed as previously described, utilizing the BepiPred web server with the FASTA sequence of *L. infantum* (syn. *L. chagasi*—GenBank: GQ290460) [9]. Three different versions of the VQ34 peptide (VGPLSVGPQSVGPLSVGPLSVGPQAVGPLSVGPQ) were commercially synthesized (GenOne Biotechnologies, Rio de Janeiro, Brazil) for use in the coupling process: unconjugated VQ34, VQ34 conjugated to BSA (VQ34-BSA), and VQ34 conjugated to a beta-alanine–lysine (x4)–cysteine peptide tag (VQ34-TAG) [29]. For BSA conjugation the VQ34 peptide was synthesized with an additional cysteine residue at the N-terminus and subsequently covalently coupled to BSA [27,28], whereas the TAG sequence was co-synthesized with the VQ34 peptide at its C-terminal end [29]. Each formulation was individually coupled to 5.5 μm polystyrene nonmagnetic carboxylated beads (COR) (Bangs Laboratories Inc, Fishers, IN, USA).

*L. infantum* total antigen (LiAg)—*L. infantum* (MHOM/BR/1974/PP75) stationary phase culture promastigotes were submitted to 10 cycles of freezing and thawing (−196 and 37 °C, respectively) and ultrasonication (40 W/15 min) in phosphate-buffered saline (PBS) (GIBCO, Billings, MT, USA) pH7.2. Final protein concentration was adjusted to 1 mg/mL. Aliquots were stored at −80 °C until beads coupling.

### 2.4. Coupling

The QuantumPlex^®^ Carboxyl 5.5 μm (5 dye intensities) kit from Bangs Laboratories Inc. (USA) was used, consisting of five groups of non-magnetic microspheres, differentiated by the intensity of their internal fluorescence with StarfireRed^®^. These microspheres have a uniform carboxyl surface, allowing for the coupling of the desired analyte. Each microsphere was coupled with one of the antigens as follows (Canine IgG and LiAg bead #1, BSA bead #2, VQ34 bead #3, VQ34-BSA bead #4, and VQ34-TAG bead #5). Antigen coupling to carboxylated beads was performed according to the protocol previously described [30] with some modifications. Beads were vortexed and sonicated for 20 s to prevent aggregation. Twenty-five microliters of beads were transferred to 2 mL microtubes (Sarsted AG & Co. KG, Nümbrecht, Germany) and were washed twice with double-distilled water (ddH_2_O), by centrifugation at 1000× *g* for 10 min each time. After removing the supernatants, 80 μL of activation buffer [0.1 M NaH_2_PO_4_·H_2_O (pH 6.2)] along with 10 μL of N-hydroxysulfosuccinimide (sulfo-NHS; Thermo Fisher Scientific, Waltham, MA, USA) and 10 μL of 1-ethyl-3-(3-dimethylaminopropyl)-carbodiimide hydrochloride (EDC; Thermo Fisher Scientific), both at a concentration of 50 mg/mL in ddH_2_O, were added. The beads were mixed with a pipette, left to incubate at room temperature for 10 min, mixed again, and then incubated for an additional 10 min. Following a total incubation period of 20 min, the beads were washed twice with a coupling buffer [PBS (GIBCO USA) pH7.2] at 1000× *g* for 10 min. Next, 20 μg of each antigen, diluted in 100 μL of coupling buffer, were introduced. After 2 h of incubation on a seesaw shaker at room temperature, the beads underwent three washes with a wash buffer [PBS pH7.2, 1% BSA, 0.02% Tween 20, 0.05% sodium azide (NaAz)]. Following another centrifugation, the beads were re-suspended in a storage buffer (PBS pH7.2, 1% BSA, 0.02% Tween 20, 10% sodium azide). The bead concentration was adjusted to 2 × 10^6^ beads/mL in the storage buffer using an automatic counter (TC20TM BIO-RAD, Hercules, CA, USA).

The same protocol was employed for coupling BSA or total canine immunoglobulin G (canine IgG) (BIO-RAD) to the beads. Total canine IgG was used as both the negative control (without serum and conjugate) and the positive control (without serum and with conjugate) in the serological reactions.

### 2.5. Serological Reaction

First, each coupled beads solution (LiAg, canine IgG, BSA, VQ34, VQ34-BSA, and VQ34-TAG) were vortexed to prevent clumping. Five microliters (10,000 beads) of each peptide-bead solution were transferred to 2 mL microtubes, and 95 μL of wash buffer was added. Serum samples from dogs, including those with clinical symptoms and asymptomatic for CVL, those vaccinated against CVL, or healthy controls, were added at a final dilution of 1:1600 in wash buffer, making a total volume of 100 µL. In the case of the IgG-bead controls, they only received the wash buffer at this stage (both negative and positive controls). Following a 15-min incubation at 37 °C on a seesaw shaker, 400 µL of wash buffer was added, and the samples were centrifuged for 10 min at 1000× *g*. The serological reaction was assessed using a fluorescein-conjugated anti-dog antibody (anti-IgG-FITC) (BIO-RAD) that was added to the samples at a 1:400 dilution in a wash buffer. The negative IgG control, on the other hand, received only the wash buffer instead of anti-IgG-FITC. After another 15-min incubation at 37 °C on a seesaw shaker, the samples were then centrifuged at 1000× *g* for 10 min, the supernatants were removed, and the beads were resuspended in 100 µL of PBS for acquisition in the flow cytometer.

### 2.6. Flow Cytometry

**Figure 1:**
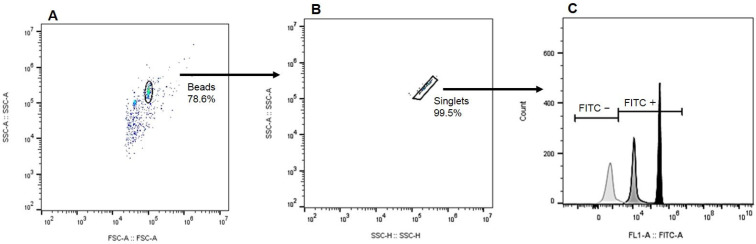
Gating strategy employed for coupling analysis: (**A**) Bead population delineation based on size (FSC-A) and granularity (SSC-A), (**B**) Subsequent exclusion of doublets to refine analysis, and (**C**) A representative histogram depicting the intensity of FITC fluorescence, with the negative control (light gray), a canine representative from the CVL+ symptomatic group (gray), and the IgG positive control (black) for comparison.

The samples were acquired using a CytoFLEX flow cytometer (Beckman Coulter, Hialeah, FL, USA) at the Oswaldo Cruz Institute Flow Facility, and the acquisition protocol was executed with the CytExpert acquisition software Version 2.4.0.28 (Beckman Coulter, Brea, CA, USA). The FITC emission from the anti-IgG-FITC conjugate was measured using a 525/40 BP filter with a 488 nm laser. The acquisition was set to stop after detecting two thousand events in the bead gate on the dot plot of Forward Scatter-Area (FSC-A) versus Side Scatter-Area (SSC-A). Analyses were performed using FlowJo 10 (Becton Dickinson, Franklin Lakes, NJ, USA) and were based on two dot plots. First, the FSC-A versus SSC-A plot was used to define the bead population and gate. Second, the Side Scatter-Height (SSC-H) versus SSC-A dot plot was employed to establish the singlet’s gate, excluding doublets from the analysis. A FITC histogram was then generated based on the gates for beads and singlets. For data analysis, we utilized the percentage of positive beads for FITC and the median fluorescence intensity as parameters (Figure 1).

### 2.7. ELISA for VQ34 Specific IgG

The IgG binding to the synthetic peptides VQ34-BSA and VQ34-TAG were measured by ELISA. Maxisorp 96-well plates (Nunc, Rochester, NY, USA) were sensitized with either VQ34-BSA or VQ34-TAG conjugated peptides individually (20 μg/mL) in PBS and conditioned overnight at 4 °C. Plates were washed with PBS and blocked with PBS-Tween containing 5% nonfat dry milk (PBS-Tween-M) for 2 h at 37 °C. Individual plasma samples were added and the plates incubated at 37 °C for 1 h. After three washes with PBS-Tween, bound antibodies were detected with the addition of goat anti canine IgG peroxidase labeled antibody (Bethyl Laboratories Inc.—Montgomery, TX, USA) diluted 1:20,000 on PBS-Tween containing 2.5% nonfat dry milk, followed by TMB (Scienco, Lages, SC, Brazil). The absorbance was obtained at 450 nm using an ELISA reader (Spectramax 250, Molecular Devices, Sunnyvale, CA, USA). Serum samples were tested in a 1:100 dilution using PBS-Tween containing 2.5% nonfat dry milk. The cut-off values for VQ34-TAG and VQ34-BSA were calculated separately, based on the mean plus 2 standard deviations of the OD values obtained from the unvaccinated control group.

### 2.8. Statistical Analysis

Statistical analyses were performed using Prism version 9 (GraphPad Software^®^, Boston, MA, USA). The normality of the distribution of variables was assessed using the Shapiro-Wilk test. The Mann-Whitney *t* test was used for comparisons between groups. The results were considered significant when *p* ≤ 0.05 with a 95% confidence interval

## 3. Results

### 3.1. Peptide TAG Enhances the Sensitivity of VQ34 in the SAT Assay, Whereas BSA Significantly Amplifies Nonspecific Responses

**Figure 2:**
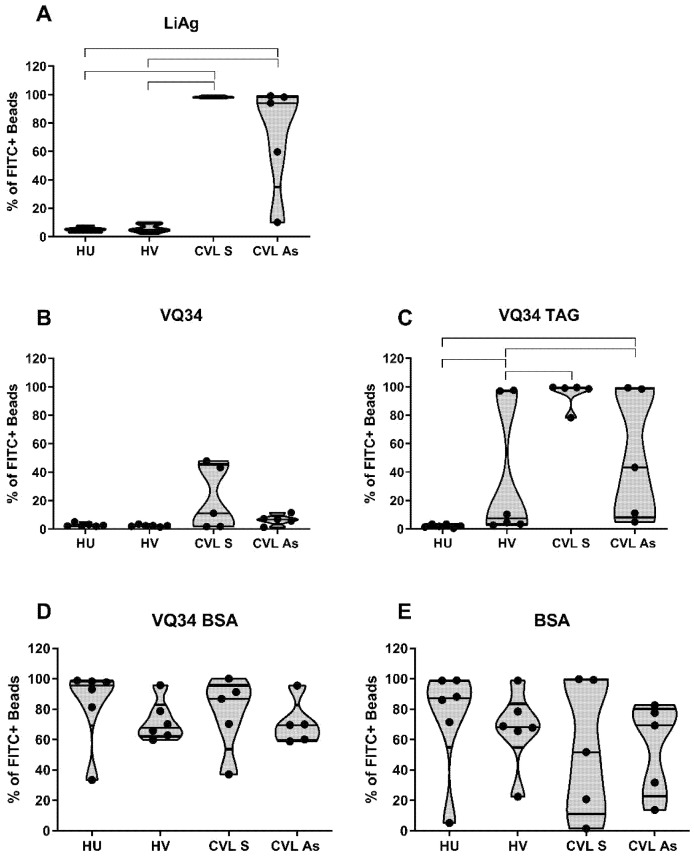
Comparative results of the percentages of FITC-positive beads coupled to *L. infantum* promastigotes total antigen ((**A**)—LiAg) and to the VQ34 peptide in three formulations: unconjugated ((**B**)—VQ34), conjugated to a beta-alanine–lysine (x4)—cysteine peptidic tag ((**C**)—VQ34-TAG), or conjugated to BSA ((**D**)—VQ34-BSA), along with beads conjugated to the BSA protein alone ((**E**)—BSA). The SAT reactions were conducted with serum samples from four groups of dogs: healthy unvaccinated (HU), healthy vaccinated (HV), CVL+ symptomatic (CVL S), and CVL+ asymptomatic (CVL As). Anti-IgG-FITC conjugate was used at a final dilution of 1:400, and serum samples at a final dilution of 1:1600. Lines connect statistically significant results with Mann-Whitney *t* test *p* < 0.05.

Our results with the total LiAg coated beads demonstrated a negative reaction to LiAg in the sera of healthy dogs, regardless of vaccination status. Conversely, all symptomatic dogs displayed high labeling percentages (above 97%), whereas a more diverse response was observed in the asymptomatic group (ranging from 10.2% to 99%) (Figure 2A).

Upon comparing the results obtained with VQ34 in its three formulations, it was evident that the VQ34-TAG displayed enhanced sensitivity, without altering the responses observed in the control group, when compared to the unconjugated VQ34 formulation (Figure 2C and Figure 2B respectively). Considering that the Leish-Tec^®^ vaccine utilizes the A2 protein as the immunogen, and that the VQ34 peptide was predicted within the same protein, the detection of a positive serological reaction in the vaccinated animal group, as well as in the groups of animals with LVC, was expected. However, unexpectedly, the use of the VQ34-BSA conjugated peptide demonstrated high staining percentage in the serological reaction across all tested groups, including the healthy unvaccinated control group, indicating a loss of specificity in the reaction (Figure 2D).

### 3.2. Assessment of IgG Levels by ELISA Confirms the Non-Specific Response Observed with the VQ34-BSA Formulation

To make sure that the lack of specificity observed with the VQ34-BSA formulation was not due to the SAT technique, we performed an ELISA assay to evaluate the IgG response in the same sera previously tested by SAT, using VQ34-TAG and VQ34-BSA formulations.

**Figure 3:**
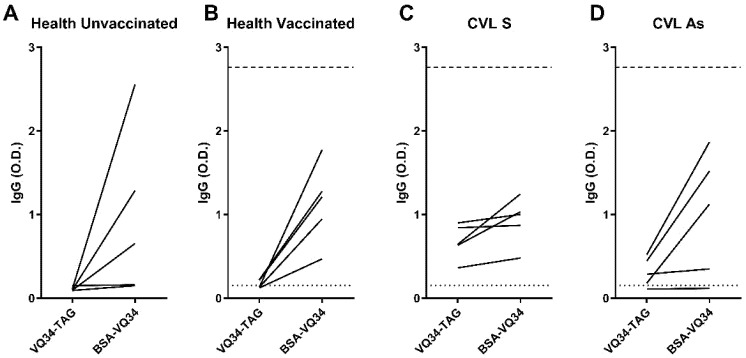
Anti-VQ34-TAG and VQ34-BSA IgG antibodies were evaluated via ELISA in the sera of 5 dogs from each studied group: (**A**) healthy unvaccinated, (**B**) healthy vaccinated, (**C**) CVL+ symptomatic (CVL S), and (**D**) CVL+ asymptomatic (CVL As). Lines connect the IgG titers obtained with the same sample for each peptide formulation. Absorbance was measured at 450 nm. The dotted line indicates the cut-off for VQ34-TAG, while the dashed line represents the cut-off for VQ34-BSA. Both cut-offs were calculated based on the mean plus 2 standard deviations of the OD values for each antigen in the unvaccinated control group.

As can be seen in Figure 3, the IgG response observed to VQ34-BSA antigen showed a notable increase compared to the response observed with VQ34-TAG formulation in the majority of sera, across all tested groups (100% of the samples in both healthy groups (Figure 3A,B) and 30% in CVL groups (Figure 3C,D). Furthermore, the observed increase in IgG response within the unvaccinated control group could significantly impact the interpretation of the results. Based on the cut-off established using the VQ34-BSA formulation, none of the samples from the tested groups were classified as positive. In contrast, the results obtained with the VQ34-TAG formulation were considerably more consistent, yielding data that align with previously published findings in the literature [9].

## 4. Discussion

In the pursuit of developing more precise diagnostic tests, the utilization of peptides identified through “in silico” prediction methodologies has proven to be an efficient and cost-effective strategy for selecting candidate antigens for disease diagnosis or vaccination [31]. Building on this approach, a recent study by our team identified linear epitopes derived from the A2 protein of *L. infantum*, which demonstrated distinct recognition profiles when exposed to serum from dogs with Canine Visceral Leishmaniasis (CVL) or those vaccinated with the Leish-Tec^®^ vaccine, as assessed by ELISA [9]. In the present study, we employed one of these predicted peptides to develop a pilot SAT-based test (Cytometric Bead Array—CBA) for diagnosing CVL. Peptide VQ34 was utilized for coupling with non-magnetic beads in three different formulations: unconjugated, conjugated with BSA, and conjugated with a beta-alanine–lysine (x4)—cysteine tag. Our preliminary results indicate that the multiplex approach may serve as an effective tool for rapid serological testing for CVL, especially by using various tag-conjugated peptides to improve detection rates. We are using different antigens, increasing the number of animals per group, and incorporating serum samples from dogs with other diseases to assess the assay’s sensitivity and reproducibility.

Multiplex assays by flow cytometry, such as CBA, offer the advantage of simultaneously analyzing and quantifying multiple analytes in small sample volumes, using standard clinical flow cytometers [32]. Furthermore, they require less time compared to ELISA [33], while maintaining accuracy and sensitivity. This approach can also be customized to detect different disease stages and utilize antigens from various diseases in a single analysis [29]. Promising results have been reported using this methodology for diagnosing malaria and cutaneous leishmaniasis [27,28,29,34,35].

Since prior findings indicated that peptides with a molecular weight below 3000 g/mol poorly couple to the beads used in SAT-type assays [27,28], we selected the VQ34 peptide for our experiments, which has an approximate molecular weight of 3134,58 g/mol [9]. However, as its weight approaches the suggested limit for effective coupling, we evaluated the VQ34 peptide not only directly coupled to the beads but also conjugated to BSA protein [27,28] or a peptide tag composed of beta-alanine-lysine (x4)-cysteine [29]. BSA increases the peptide’s molecular weight, while the linkage of the peptide to the COOH group of the bead is facilitated by the four lysine residues present in the “TAG”, thereby improving coupling efficiency [29].

The evaluation of the VQ34 peptide in various formulations yielded intriguing and unforeseen results. Despite its molecular weight slightly surpassing the threshold previously described for efficient coupling with the beads, we observed heightened and more consistent staining when the VQ34-TAG was utilized in the reactions, in comparison to those obtained with the VQ34 formulation. Our results using the VQ34-TAG in the SAT assay closely mirrored our previous findings with the VQ34 peptide using ELISA [9], demonstrating the importance of the TAG for achieving more efficient conjugation of the antigen to the beads. The most pronounced responses to the VQ34-TAG formulation were observed in the symptomatic CVL+ group showing significantly higher responses when compared to health vaccinated or unvaccinated animals (Mann Whitney *t* test *p* = 0.0173 and *p* = 0.0043 respectively). In contrast, the healthy unvaccinated animals exhibited only a baseline response. Two animals from the vaccinated group exhibited a significant percentage of staining for VQ34-TAG peptide using the SAT technique. Although able to discriminate between symptomatic animals and healthy animals, regardless of their vaccination status, VQ34-TAG could not differentiate the asymptomatic group from the vaccinated group. As previously reported, this is not an unexpected finding, since the animals were immunized with the Leish-Tec^®^ vaccine, whose immunogen is the A2 protein, from which the VQ34 peptide is derived [9].

Although our current findings with the VQ34-TAG formulation yielded results consistent with previous reports, the VQ34-BSA formulation produced conflicting results. In this case, all tested groups exhibited high staining percentages, contrary to previous data from our group and those currently obtained with the VQ34 and VQ34-TAG formulations, which indicates nonspecific responses, sometimes higher in the serum of healthy, unvaccinated animals. However, the inclusion of control beads conjugated with BSA was essential to demonstrate that this lack of specificity is associated with BSA rather than the peptide itself. Our interpretation is that this phenomenon arises from the presence of anti-BSA antibodies in the serum of the majority of dogs analyzed, irrespective of their health status.

BSA is not indicated for therapeutic use in dogs [36] but is widely used in cell cultivation for vaccine production in animals, and as a stabilizer in immunogens [37]. Consequently, it is possible to detect anti-BSA antibodies in healthy dogs [38]. Supporting this hypothesis, Hogenesch and colleagues [39] observed a notable presence of anti-BSA antibodies for up to 50 weeks in dogs following rabies vaccination. Additionally, the development of anti-BSA antibodies may be associated with changes in intestinal mucosal permeability in dogs with chronic enteritis [40]. Anti-BSA antibodies have also been implicated in food allergies [41] and arthritis [42] in dogs.

By utilizing *L. infantum* promastigotes total antigen (LiAg) as a control in the assay, we were able to confirm that the serological responses of the analyzed dog groups yielded the expected results, in accordance with numerous previous findings in the literature. It was observed that symptomatic dogs displayed the greatest anti-LiAg response, followed by the asymptomatic group, both showing significant differences for the healthy groups (vaccinated or not—Mann Whitney *t* test *p* < 0.005), which did not respond, demonstrating that the SAT methodology used in the current study is promising and that LiAg remains a potential marker for infected animals. On the other hand, the heterogeneous responses observed in the asymptomatic CVL group is well documented in the literature, with animals presenting different levels of antibodies, often presenting a negative reaction [43]. Quinnell and collaborators [44] demonstrated that sick dogs had the highest levels of all IgG subclasses evaluated, when compared to healthy animals. Another study also found higher total IgG titers in symptomatic dogs compared to asymptomatic dogs and CVL negative animals [45].

Our findings using LiAg and VQ34-TAG coupled beads clearly illustrate the potential of the multiplex approach as a rapid and sensible serological test for CVL. Conjugation of peptides with the TAG significantly enhanced the serological response compared to peptides used alone. However, the SAT reaction performed with BSA-conjugated peptides revealed the presence of anti-BSA antibodies in the serum of animals from all groups, including the control group. This indicates that BSA should be avoided as a carrier in bead coupling assays for serological tests with canine samples, due to its detrimental effect on test specificity and sensitivity.

## Data Availability

The raw data supporting the conclusions of this article will be made available by the authors on request.
